# IL-18 and S100A12 Are Upregulated in Experimental Central Retinal Vein Occlusion

**DOI:** 10.3390/ijms19113328

**Published:** 2018-10-25

**Authors:** Lasse Jørgensen Cehofski, Anders Kruse, Svend Kirkeby, Alexander Nørgård Alsing, Jonas Ellegaard Nielsen, Kentaro Kojima, Bent Honoré, Henrik Vorum

**Affiliations:** 1 Department of Ophthalmology, Aalborg University Hospital, Hobrovej 18-22, 9000 Aalborg, Denmark; anders.kruse@rn.dk (A.K.); noergaard.alsing@rn.dk (A.N.A.); Henrik.vorum@rn.dk (H.V.); 2 Biomedical Research Laboratory, Aalborg University Hospital, 9000 Aalborg, Denmark; 3 Department of Clinical Medicine, Aalborg University, 9000 Aalborg, Denmark; 4 Department of Odontology, School of Dentistry, University of Copenhagen, 1017 Copenhagen, Denmark; skir@sund.ku.dk; 5 Department of Clinical Biochemistry, Aalborg University Hospital, 9000 Aalborg, Denmark; j.elleggaard@rn.dk; 6 Department of Ophthalmology, Kyoto Prefectural University of Medicine, 6028566 Kyoto, Japan; kenkojim@koto.kpu-m.ac.jp; 7 Department of Biomedicine, Aarhus University, 8000 Aarhus, Denmark; bh@biomed.au.dk

**Keywords:** retina, retinal vein occlusion, proteomics, mass spectrometry, interleukin, IL-18, S100A12, annexin, complexin

## Abstract

Retinal vein occlusion (RVO) is a common retinal vascular disease. RVO may be complicated by pronounced ischemia that often leads to severe loss of visual function. The present work aimed at studying the retinal proteome of RVO complicated by ischemia. In six Danish Landrace pigs RVO was induced with argon laser in the right eye of each animal. As four retinal veins were occluded, the RVO best corresponded to a central retinal vein occlusion (CRVO). Left control eyes received a similar laser treatment without inducing occlusion. RVO and retinal ischemia were verified by angiography. The retinas were collected 15 days after RVO for proteomic analysis. RVO resulted in a downregulation of proteins involved in visual perception, including rhodopsin, transducin alpha chain, and peripherin-2. RVO also caused a downregulation of proteins involved in neurotransmitter transport, including glutamate decarboxylase 1 (GAD1), glutamate decarboxylase 2 (GAD2), and complexins 2–4. RVO lead to increased contents of proteins involved in inflammation, including interleukin-18 (IL-18), S100A12, and annexin A1 (ANXA1). Immunohistochemistry revealed a general retinal upregulation of IL-18 and ANXA1 while S100A12 was highly abundant in retinal ganglion cells in RVO. IL-18 and S100A12 are likely to be driving forces in the inflammatory response of RVO complicated by ischemia. Our findings also suggest that RVO results in compromised neurotransmission and a downregulation of proteins involved in visual perception.

## 1. Introduction

Retinal vein occlusion (RVO) is a potentially sight-threatening condition and one of the most common vascular diseases of the retina [1]. RVO may be complicated by retinal ischemia that is associated with a poor prognosis and severe loss of visual function [1,2]. Ischemia in the retina also promotes other sight-threatening complications of RVO, such as macular edema, neovascularization, and neovascular glaucoma [1,3]. RVO complicated by ischemia may also be resistant to modern treatments, such as intravitreal anti-vascular endothelial growth factor (VEGF) agents and dexamethasone intravitreal implants [4,5]. Ischemia in RVO is known to potentiate the upregulation of VEGF, VEGF receptor-2, platelet-derived growth factor and the inflammatory response that contributes to vision loss in RVO [6,7,8]. The retinal proteome of RVO complicated by ischemia remains largely unstudied. Studying the retinal protein profile in RVO complicated by ischemia may help identify novel therapeutic targets and bring important insights into the pathological processes that take place in RVO. In the present study, we report on large-scale protein changes in an experimental model of RVO that develops retinal ischemia.

## 2. Results

### 2.1. Experimental Retinal Vein Occlusion (RVO)

**Figure 1:**
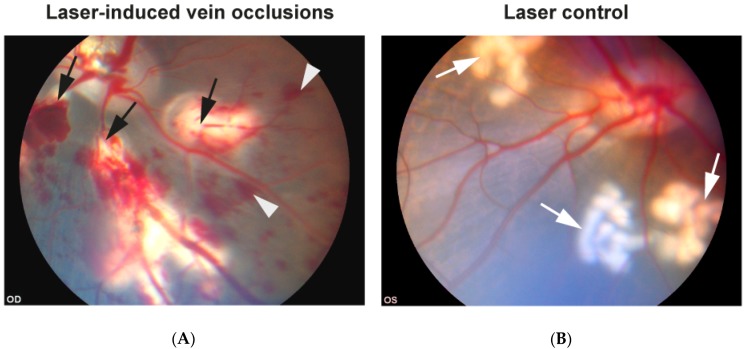
Funduscopic photos taken within 20 min after experimental retinal vein occlusion (RVO) was induced. (**A**) Eye with experimental RVO. By occluding four retinal veins a condition corresponding to central retinal vein occlusion (CRVO) was induced. Laser-induced occlusion resulted in dilation of the occluded vessels upstream of the occlusion sites. Flame-shaped hemorrhages developed shortly after vein occlusions were induced. Black arrows: Laser-induced occlusions. White arrow heads: Flame-shaped hemorrhages. (**B**) Control eye. Areas of laser applications were similar to the RVO eye. By ensuring that the laser burns in the control eye did not hit any vessels, no occlusions were induced. White arrows: Laser burns given without inducing occlusion.

**Figure 2:**
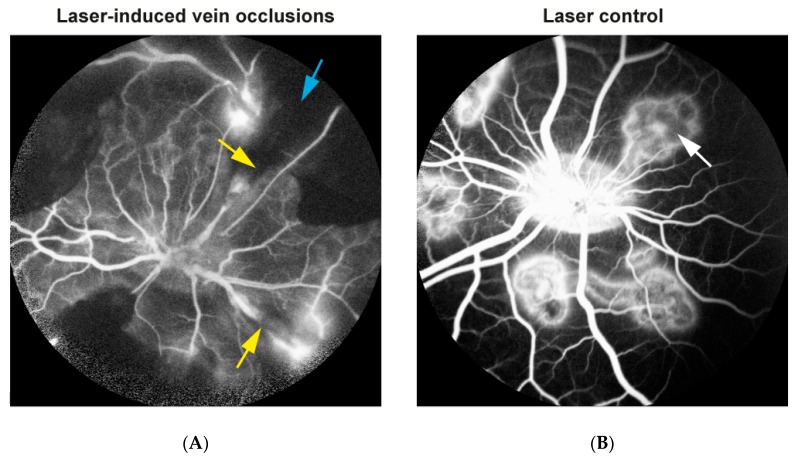
Fluorescein angiography. Angiography conducted five days after RVO. (**A**) Eye with RVO. Angiography revealed that no re-canalization of the occlusions occurred. Retinal ischemia was seen as dark areas of retinal non-perfusion. Yellow arrows: Sites of occlusion. Blue arrow: Retinal ischemia. (**B**) Control eye. Laser burns were created without inducing RVO. No retinal ischemia was observed. White arrow: Retinal changes after laser applications.

RVO was successfully induced in the right eye of each animal based on stagnation of venous blood and development of flame-shaped hemorrhages (Figure 1A). Control laser was given in the left eyes without inducing occlusion (Figure 1B). Angiography performed five days after RVO showed no re-canalization in RVO eyes (Figure 2A). Angiography also revealed that eyes with RVO developed ischemia upstream of the occluded veins (Figure 2A).

### 2.2. Quantification with Tandem Mass Tags (TMT) Based Proteomics

**Figure 3:**
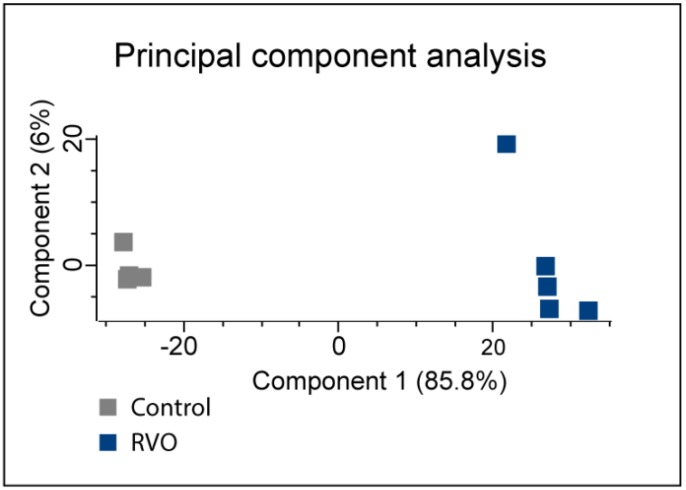
Principal component analysis (PCA). The PCA plot revealed that retinas with ischemic RVO could be separated from laser controls based on proteome changes.

A total of 3791 proteins were successfully identified and quantified in all samples (Supplementary File S1). Unfiltered mass spectrometry data is provided in Supplementary File S2. A statistically significant change was observed in 147 proteins following experimental ischemic RVO. Among these proteins, 106 proteins were upregulated and 41 proteins were downregulated. The entire list of significantly changed proteins is given in Supplementary File S3. The principal component analysis (PCA) plot revealed that the proteome of ischemic RVO was highly different from laser controls (Figure 3).

**Figure 4:**
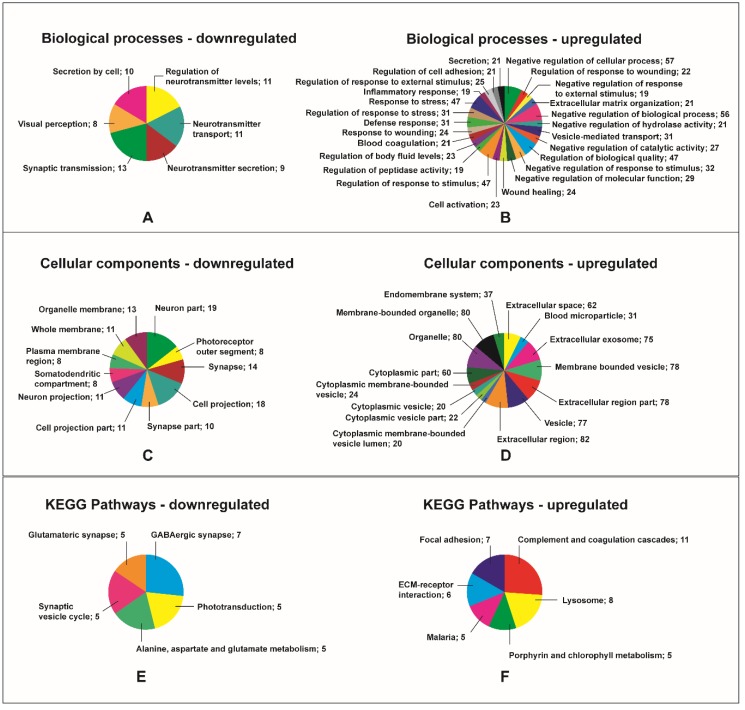
Gene Ontology analysis of differentially regulated proteins. Downregulated and upregulated proteins were analyzed as two individual groups. Numbers refer to the number of proteins that represented a given process, compartment, or pathway. (**A**) RVO resulted in a downregulation of proteins involved in visual perception. Downregulated biological processes also included processes related to neurotransmitter regulation and neurotransmitter transport as well as synaptic transmission. (**B**) Upregulated biological processes that were upregulated in RVO included response to wounding, wound healing, blood coagulation, and response to wounding. Nineteen upregulated proteins were involved in the inflammatory response in RVO (see also Table 3). (**C**) The analysis of cellular components indicated a downregulation of proteins pertaining to neuron part, photoreceptor outer segment, and neuron projection. (**D**) Gene ontology analysis of upregulated cellular components indicated that ischemic RVO causes extracellular changes. RVO resulted in an upregulation of extracellular space, extracellular exosome, and extracellular region. (**E**) Downregulated Kyoto Encyclopedia of Genes and Genomes KEGG pathways included GABAergic synapse, phototransduction, and glutamateric synapse. (**F**) KEGG pathways that were upregulated in ischemic RVO included complement and coagulation cascade, lysosome, and focal adhesion.

The bioinformatic analyses revealed that upregulated and downregulated proteins were involved in very different biological functions (Figure 4A–F).

### 2.3. Experimental RVO Results in Downregulation of Visual Perception and Neurotransmitter Transport

**Figure 5:**
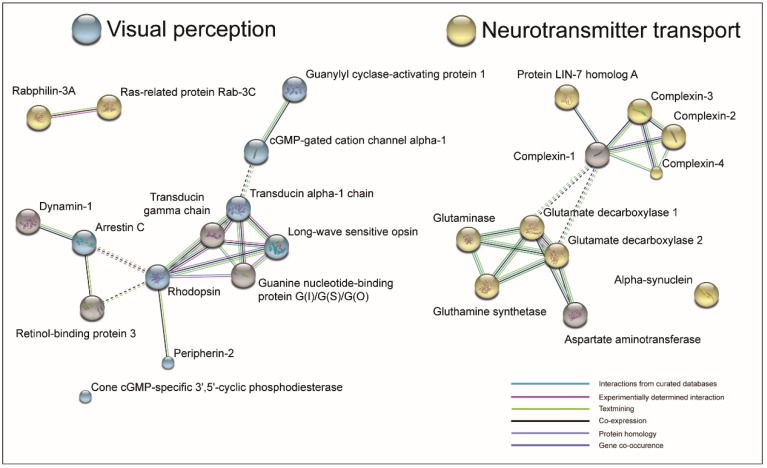
Cluster analysis of downregulated proteins with interactions. Proteins that were downregulated in ischemic RVO grouped into at least three clusters. One cluster was centered around photoreceptor proteins, such as rhodopsin, transducin alpha-1, and transducin gamma chain. A second cluster consisted of glutamate decarboxylase 1 (GAD1), glutamate decarboxylase 2 (GAD2), glutaminase, glutamine synthetase, and aspartate aminotransferase. The third cluster consisted of complexins 1–4 and protein LIN-7 homolog A. Gene ontology analysis showed that proteins in the cluster of photoreceptor proteins were predominantly involved in visual perception (blue color) whilst complexins and proteins involved in glutamate metabolism were involved in neurotransmitter transport (yellow color).

RVO resulted in a downregulation of proteins involved in visual perception (Figure 4A). RVO also lead to a downregulation of proteins involved in neurotransmitter transport, regulation of neurotransmitter levels, neurotransmitter secretion, and synaptic transport (Figure 4A). RVO was associated with decreased levels of proteins pertaining to photoreceptor outer segment, neuron part, and neuron projection (Figure 4C). Pathway analysis revealed a downregulation of GABAergic synapse and glutamateric synapse in RVO (Figure 4E). Downregulated proteins clustered into at least three groups (Figure 5). Downregulated clustering proteins were involved in visual perception and neurotransmitter transport (Figure 5).

Proteins involved in visual perception included rhodopsin, peripherin-2, arrestin C, cone cGMP-specific 3′,5′-cyclic phosphodiesterase (PDE6C), transducin alpha subunit, long-wave sensitive opsin, isoform 2 of cGMP-gated cation channel alpha-1 (CNGA1) and guanylyl cyclase-activating protein 1 (GCAP1) (Figure 5, Table 1).

**Figure 6:**
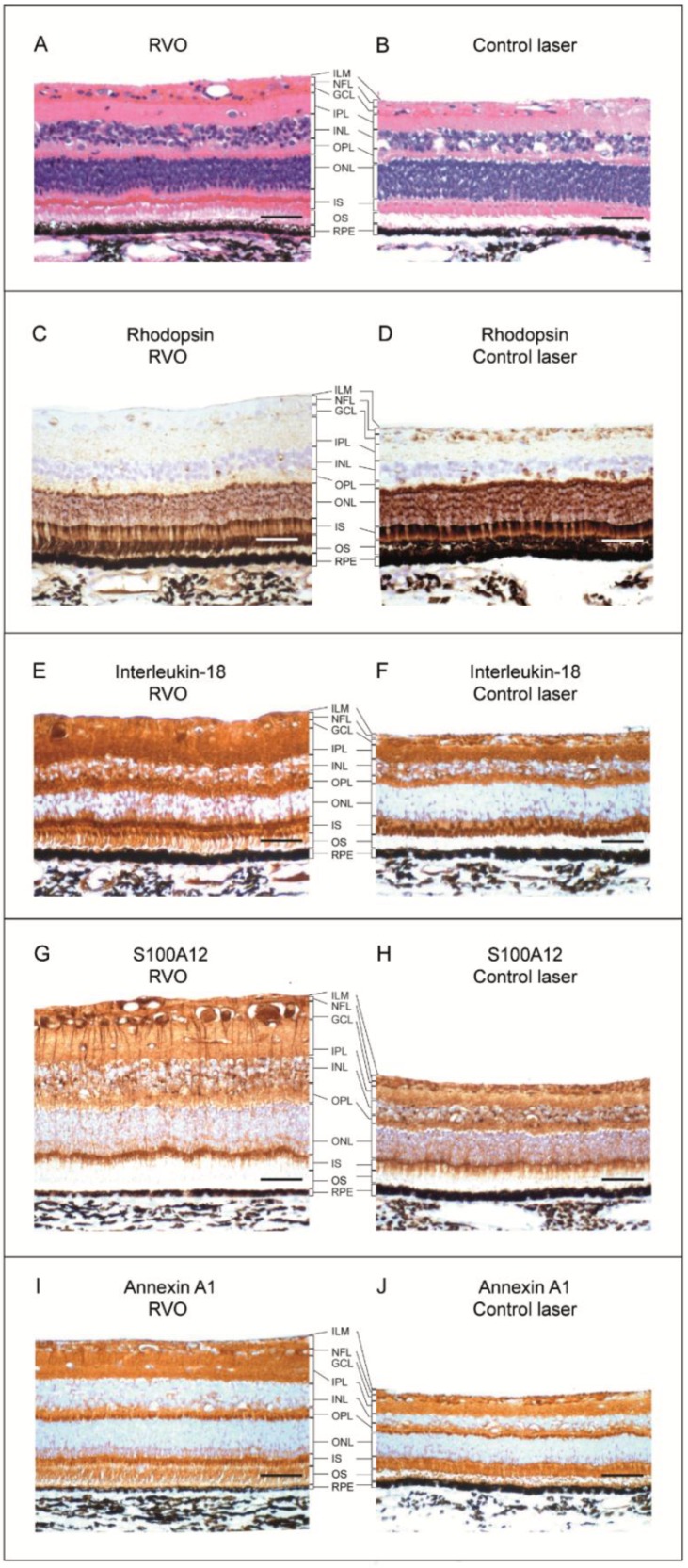
Histology and immunohistochemistry. Scale bar = 31 µm. Reaction color: brown. (**A**,**B**) Hematoxylin and eosin (HE) staining. In RVO complicated by ischemia, increased thickness was observed in the nerve fiber layer (NFL), the ganglion cell layer (GCL), the inner plexiform layer (IPL), and the outer plexiform layer (OPL). (**C**,**D**) In RVO, rhodopsin was downregulated in the outer nuclear layer (ONL), the photoreceptor inner segments (IS), and outer segments (OS). (**E**,**F**) Immunohistochemistry confirmed a general upregulation of IL-18 in ischemic RVO. (**G**,**H**) S100A12 was abundant in retinal ganglion cells in RVO. (**I**,**J**) ANXA1 was abundant in the inner retinal layers in RVO.

Immunohistochemistry revealed a general retinal thickening in RVO compared to controls (Figure 6A,B) and confirmed a downregulation of rhodopsin in RVO (Figure 6C,D). Downregulated clustering proteins involved in neurotransmitter transport included protein LIN-7 homolog A, complexins 2–4, glutamate decarboxylase 1 (GAD1), glutamate decarboxylase 2 (GAD2), glutaminase, glutamine synthetase, and aspartate aminotransferase (Figure 5, Table 2).

### 2.4. Blood Coagulation, Focal Adhesion, and Inflammatory Response are Upregulated in RVO

RVO was followed by an upregulation of response to wounding, blood coagulation, wound healing, and response to stress (Figure 4B). RVO also activated cell adhesion and extracellular matrix organization (Figure 4B). Furthermore, ischemic RVO was associated with an upregulation of proteins involved in the inflammatory response, including interleukin-18 (IL-18), S100A12, and annnexin A1 (ANXA1) (Figure 4B, Table 3). RVO also resulted in increased contents of proteins pertaining to extracellular space, extracellular exosome, extracellular region part, extracellular region, and extracellular matrix (Figure 4D). RVO also caused an upregulation of proteins associated with vesicles (Figure 4D). Pathway analysis revealed that ischemic RVO was associated with increased levels of proteins involved in complement and coagulation cascades, extracellular matrix-receptor interaction, and focal adhesion (Figure 4F). RVO resulted in an upregulation of clustering plasma proteins (Supplementary File S4). These plasma proteins were involved in blood coagulation and included fibrinogen chains, coagulation factors, apolipoproteins, prothrombin, antithrombin-III, and serum albumin (Supplementary File S5).

### 2.5. Inflammation in RVO-Interleukin-18 (IL-18), S100A12, and Annexin (ANXA1)

Nineteen proteins involved in inflammatory response were increased in content in ischemic RVO (Table 3). Proteins involved in inflammatory response included scavenger receptor cysteine-rich type 1 (CD163), ANXA1, IL-18, S100A12, and lysozyme c-2 (Table 3). Furthermore, a number of plasma proteins were identified to be involved in the inflammatory response (Table 3). As inflammation contributes to visual impairment in RVO, the inflammatory proteins, IL-18, S100A12, ANXA1 and fibronectin, (Table 3) were selected for further validation.

**Figure 7:**
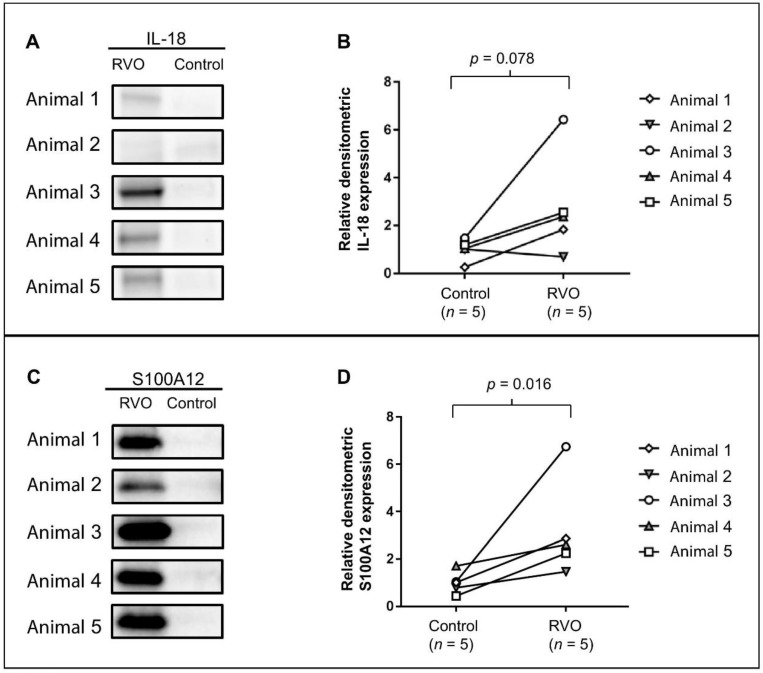
Western blot analysis of IL-18 and S100A12. (**A**) Protein content of IL-18 (approximately 22 kDa). (**B**) Densitometric data presented as the change of relative IL-18 expression between ischemic RVO and control samples, normalized to relative β-actin expression. Western blot analysis supported the data from mass spectrometry, though not statistically significant (*p* = 0.078). (**C**) Protein content of S100A12. (**D**) Densitometric data confirmed an upregulation of S100A12 in ischemic RVO (*p* = 0.016).

Mass spectrometry revealed a seven-fold upregulation of IL-18 in ischemic RVO (Table 3). Western blotting confirmed the upregulation of IL-18 (Figure 7A,B). Immunohistochemistry confirmed a general upregulation of IL-18 in ischemic RVO compared to laser control (Figure 6E,F).

RVO resulted in a five-fold upregulation of S100A12, which was confirmed by Western blotting (Figure 7C,D). In ischemic RVO, S100A12 was particularly abundant in retinal ganglion cells and their dendritic processes (Figure 6G).

Immunohistochemistry confirmed an upregulation of ANXA1, which was abundant in layers with increased thickness, such as the nerve fiber layer, the ganglion cell layer, the inner plexiform layer, and the outer plexiform layer (Figure 6I). Proteomic analysis revealed a strong upregulation of fibronectin. However, the upregulation of fibronectin was not confirmed by immunohistochemistry (Supplementary File S6).

## 3. Discussion

### 3.1. Experimental Retinal Vein Occlusion (RVO)

In this study, four retinal veins were occluded in the RVO eye. Therefore, the condition that was induced corresponded best to a central retinal vein occlusion (CRVO). The porcine retina has no central vein that is accessible for laser induced vein occlusion [9]. Occlusion of four retinal veins was observed to be complicated by retinal ischemia. We previously conducted studies in which only one retinal vein was occluded [10,11,12,13]. Occlusion of a single retinal vein in healthy pigs does not result in retinal ischemia and retinal thickening [11,14]. However, the present study revealed that retinal thickening occurs when four retinal veins are occluded. Retinal thickening observed as macular edema is a frequent complication in humans with RVO [6,15,16]. Thus, retinal thickening, as observed in the present study, is a complication that is likely to occur. Indeed, an RVO model with occlusion of four retinal veins may be better suited for studying retinal thickening following RVO.

Based on the histological analyses, retinal thickening mainly occurred in the nerve fiber layer, the ganglion cell layer, the inner plexiform layer, and the outer plexiform layer (Figure 6). This finding is consistent with the fact that retinal veins drain the inner two thirds of the retina [6,9,17]. On the other hand, the morphology of the retinal pigment epithelium (RPE) remained largely unchanged (Figure 6). The RPE is not likely to be affected in RVO as the RPE is drained by the choroid.

### 3.2. Quantification by Mass Spectrometry

Protein studies of RVO have mainly focused on a limited number of specific proteins [10]. Our study revealed a statistically significant change in more than 100 proteins following ischemic RVO. Thus, our data indicate that pathological changes in RVO are driven by alterations in multiple proteins rather than a few vasoactive proteins.

The PCA plot (Figure 3) revealed that the proteome of ischemic RVO was highly different from laser controls. Similar differences may be observed in a clinical setting. Indeed, the proteome of an eye with ischemic RVO is likely to be highly different from a healthy fellow eye. Thus, a patient may have severe loss of visual function in one eye due to ischemic RVO whilst the fellow eye may have normal visual acuity [1,2]. 

#### 3.2.1. Visual Perception

A number of proteins involved in visual perception were downregulated following ischemic RVO (Table 1). A large proportion of these proteins were photoreceptor proteins involved in phototransduction, including rhodopsin, long-wave-sensitive opsin 1, transducin alpha subunit, PDE6C, CNGA1, and GCAP1. Compromised function of these proteins is known to cause retinal degeneration [18,19,20]. Thus, the downregulation of these proteins in RVO may represent an early stage of retinal degeneration.

Ischemic RVO was also associated with a decreased level of peripherin-2, an integral membrane glycoprotein located in the outer segments of cone and rod photoreceptors [21,22,23]. Peripherin-2 is required for rod and cone photoreceptor outer segment formation and function [24,25], and a decreased content of peripherin-2 may affect the maintenance of photoreceptor structure.

#### 3.2.2. Neurotransmitter Transport

Downregulated proteins involved in neurotransmitter transport included the glutamate decarboxylase isoforms named GAD1 and GAD2, which catalyze the production of gamma-aminobutyric acid (GABA), the main retinal inhibitory transmitter [26,27]. Thus, inhibitory neurotransmission may be compromised in ischemic RVO due to the decreased levels of GAD1 and GAD2. The downregulation of GAD1 and GAD2 may be associated with ischemic processes as decreased levels of GAD1 and GAD2 have previously been observed with retinal ischemia [28].

Downregulated proteins involved in neurotransmitter transport also included complexins 2–4 (Table 2). Complexins are small presynaptic proteins that control synaptic vesicle fusion and prevent spontaneous neurotransmitter release [29]. Decreased levels of complexins are likely to affect synaptic vesicle fusion and neurotransmitter release.

#### 3.2.3. Inflammation in RVO

A statistically significant increase was identified in 19 proteins involved in inflammatory response (Figure 4B, Table 3). The increased content of inflammatory proteins in RVO is likely to be associated with the ischemia that was observed in the RVO model. Previous studies have demonstrated that increased levels of IL-6, IL-8, and monocyte chemotactic protein-1 (MCP-1) in RVO are closely associated with the severity of retinal ischemia [7,8,30,31]. Studies of the inflammatory response in RVO have primarily focused on a small number of cytokines. However, results from the present study indicated that numerous inflammatory proteins changed following ischemic RVO (Table 3). Inflammatory proteins that have not previously been associated with RVO included IL-18, S100A12, lysozyme C-2, and CD163.

A number of plasma proteins were also classified as proteins involved in the inflammatory response (Table 3). The biological role of these plasma proteins is difficult to interpret as the blood-retinal barrier is known to be compromised in RVO [14]. A compromised blood-retinal barrier results in an influx of plasma proteins [14]. Thus, the increased levels of plasma proteins may primarily represent a disruption of the blood-retinal barrier.

#### 3.2.4. IL-18

IL-18 is a cytokine that is produced by monocytes, glial cells, and dendritic cells [32]. IL-18 increases in content under inflammatory conditions where it promotes upregulation of other cytokines, chemokines, and adhesion molecules [33]. The increased level of IL-18 in ischemic RVO is of interest as it is not among the interleukins that are normally associated with RVO. While other cytokines, such as IL-6 and IL-8, are known to be involved in inflammatory processes in RVO [7,8,30], there are very few reports of IL-18 in RVO.

Results from animal studies indicate that IL-18 is involved in inflammation caused by ischemia. Qi et al. [34] demonstrated that retinal IL-18 was upregulated in retinal ischemia in an experimental rat model of ischemia-reperfusion. Myocardic IL-18 is also increased following myocardial ischemia-reperfusion injury [33,35]. IL-18 neutralizing antibodies have been found to reduce the size of myocardial infarction in animal studies [33].

A protective role of IL-18 has also been proposed. Shen et al. [36] found that aqueous IL-18 increased in patients with retinal vein occlusion after anti-VEGF intervention and detected a positive correlation between intraocular IL-18 and improved visual acuity. IL-18 has also been found to reduce retinal neovascularization in mice with ischemic retinopathy and to counteract VEGF-induced vascular leakage [36].

Using an exploratory design the present study aimed at identifying novel proteins associated with ischemic RVO. Additional studies operating with larger samples sizes than in our study will be necessary to further establish the role of IL-18 in ischemic RVO.

#### 3.2.5. S100A12 and ANXA1

S100A12 belongs to the S100 family of calcium binding proteins. S100A12 is secreted by activated granulocytes [37] and constitutes approximately 5% of the total cytosolic amount of proteins in neutrophil granulocytes [38,39]. S100A12 has chemotactic activity for mast cells and monocytes [40]. Serum S100A12 is a marker of inflammatory disease as well as infectious disease [39,41,42,43].

Serum S100A12 is elevated in various types of uveitis [44,45]. The present study indicates that S100A12 is also associated with inflammation following ischemic RVO and ischemia. In ischemic RVO, S100A12 was highly abundant in the cell bodies and dendritic processes of retinal ganglion cells. The expression of S100A12 in retinal ganglion cells has not previously been described and may represent local inflammation following ischemic RVO.

ANXA1 belongs to the annexin superfamily of Ca^2+^-dependent phospholipid-binding proteins. ANXA1 is an important regulator of the innate immune system. ANXA1 mediates resolution of inflammatory processes and anti-inflammatory actions of glucocorticoids [46,47]. Based on the anti-inflammatory features of ANXA1, it may be considered that ANXA1 counteracts the inflammatory response in ischemic RVO.

## 4. Materials and Methods

### 4.1. Animal Preparation

The experiments were approved by the Danish Animal Experiments Inspectorate (permission number, 2016-0201-00971, 1 July 2016). Six Danish Landrace pigs (30–40 kg) were used for the experiments. A 12-h light/dark cycle was used during the entire housing. On the day prior to the experimental procedures, the animals were fed in the morning and had access to unlimited amounts of water until the experiments were performed. Animal anesthesia was performed in accordance with Danish legislation on the care and use of laboratory animals. The procedures for animal anesthesia were approved by the Danish Animal Experiments Inspectorate (permission 2016-0201-00971). The animals were anesthesized with an intramuscular injection of Zoletil mixture consisting of ketamine 6.25 mg/mL and tiletamine 6.25 mg/mL, zolazepam 6.25 mg/mL, butorphanol 1.25 mg/mL, and xylazine 6.5 mg/mL. The dose of the Zoletil mixture was 1 mL/10 kg. Local anesthesia was performed with Oxybuprocaine Hydro 0.4% eye drops (Mydriacyl: Bausch & Lomb) and Phenylephrine 10% eye drops (Metaoxidrin; Bausch & Lomb). Dilation of the pupils was performed as previously described [10,11].

### 4.2. Experimental Vein Occlusion

Ischemic RVO was induced in the right eye of each animal while the left eye served as a control (Figure 1). Laser induced vein occlusion was induced as previously described [10,11]. Briefly, ischemic RVO was induced in the right eyes by occluding four retinal veins close to the optic nerve head with a standard argon green laser given by indirect ophthalmoscopy using a 20 diopter lens. For each occlusion, 30–40 laser applications were used. Laser applications were performed with an energy of 400 mW and an exposure time of 550 ms. Identical laser burns were made in the left control eyes without inducing occlusion by applying the laser to areas close to the optic nerve head that were devoid of any major vessels. Fluorescein angiography was conducted five days after RVO to confirm that the veins remained occluded and to confirm that retinal ischemia developed. The eyes were enucleated 15 days after RVO was induced. Enucleation was performed by surgical removal of the eyelids and adnexa. The eye ball was then removed by cutting the optic nerve without causing any damages to the optic nerve head. Immediately after enucleation, the animals were euthanized with an intravenous injection of Euthasol 400 mg/mL (Virbac Danmark A/S, Kolding, Denmark) 0.5 mL/kg.

### 4.3. Sample Preparation for Mass Spectrometry

Paired retinal samples from five pigs were used for tandem mass tags (TMT) based MS. Thus, samples with ischemic RVO (*n* = 5) were compared with laser controls (*n* = 5). The dissection was performed as described in previous works [11,12]. Briefly, eyes used for mass spectrometry were placed on ice immediately after enucleation and blood residues were removed by rinsing the eyes with cooled saline water. The eyes were kept on ice during the entire dissection, which was performed under a microscope. The anterior segment was removed and the vitreous body was gently aspired with an 18 G needle (diameter = 1.2 mm) into a 5 mL syringe. The neurosensory retina was peeled from the retinal pigment epithelium with surgical tweezers. The neurosensory retina was peeled carefully from the RPE to ensure that the RPE remained in the eye cup. Samples containing the neurosensory retina were stored at −80 °C until further use. When sample preparation for mass spectrometry was initiated, the samples were thawed and each sample was lysed with 500 µL lysis buffer consisting of 50 µL 10% SDS added to 450 µL mM triethyl ammonium bicarbonate (TEAB). The protein concentration was determined with a non-interfering assay (NI Protein Assay, Geno Technology Inc., St. Louis, MO, USA) according to the manufacturer’s instructions. In short, small volumes of the protein solutions were precipitated with the universal protein precipitating agent (UPPATM) supplied with the kit. One hundred µL of the copper solution was added to the precipitated protein. BSA was used as standard. Samples were incubated for 15–20 min. at room temperature and the absorbance at 480 nm was measured immediately after.

Reduction of disulfide bonds, alkylation with iodoacetamide, and acetone precipitation were performed as described in a previous work [13]. Digestion, TMT labeling, C18 spin column purification, and high pH reversed-phase peptide fractionation into 8 fractions were performed essentially as previously described [10].

### 4.4. Mass Spectrometry

The 8 fractions containing the peptides were resuspended in 0.1% formic acid (FA) prior to liquid chromatography mass spectrometry. Peptide concentrations of the fractions were measured by fluorescence using tryptophan as the standard and by anticipating that 0.0117 g of tryptophan corresponds to 1 g of protein, as it is the case for human and mouse proteins [48]. One microgram of each fraction (between 1 to 4.1 µL) was loaded for each run into a Dionex UltiMate^TM^ 3000 RSLC nano system coupled to an Orbitrap Fusion mass spectrometer (Thermo Scientific, Waltham, MA, USA) equipped with an EasySpray^TM^ ion source and an Easy-IC using fluoranthene as internal calibrant. The ion transfer tube temperature was 275 °C. Liquid chromatography and mass spectrometry using the TMT synchronous precursor selection MS3 mode were performed as described in detail in a recent study [13]. Briefly, peptides were separated on an Easy Spray^TM^ analytical column at 40 °C (500 mm × 75 µm PepMap RSLC, C18, 2 µm, 100 Å, Thermo Scientific, Waltham, MA, USA). The nanoflow was set to 300 µL/min. Buffers included buffer A (0.1% FA) and buffer B (80% acetonitrile, 20% water, 0.1% FA). A gradient of 240 min was applied using a gradient of buffer B from 6 to 90%. In the Orbitrap, full scans were obtained with a mass range of 380–1500 *m*/*z* at a resolution of 120,000 with an automatic gain control (AGC) of 2 × 10^5^ and maximum injection time of 50 ms. To perform MS2 acquisitions, the precursor ions were isolated with a quadrupole mass filter. MS2 acquisitions were then performed in the linear ion trap in *m*/*z* normal auto scan range mode, applying collision-induced dissociation (CID) with a collision energy of 35%, an AGC target of 1 × 10^4^, and a maximum injection time of 50 ms. A maximum of 10 precursor ions were isolated with synchronous precursor selection using and detected in MS3 in the Orbitrap in the mass range of 120–500 *m*/*z* with high energy CID using a collision energy of 65% and an AGC target of 1 × 10^5^ and a maximum injection time of 120 ms. The quantitative data obtained with TMT labelling is reporter based with isobaric tags. Data in each channel were normalized to the total peptide amount generating data from which relative fold changes of proteins can be calculated between groups.

Raw data files were processed in Proteome Discoverer 2.1. as described in our previous study [10]. Databases were downloaded in Proteome Discoverer from ProteinCenter. The Sequest HT search engine was used to search against the SwissProt *Homo sapiens* databse (SwissProt TaxID = 9606 and subtaxonomies, v2016-11-30) and the SwissProt *Sus scrofa* database (SwissProt TaxID = 9823 and subtaxonomies, v2016-11-30). For protein identification, a false discovery rate (*FDR*) < 0.01 was applied. Mass spectrometry raw data was uploaded to ProteomeXchange.

### 4.5. Statistics

The data was uploaded to Perseus version 1.6.0.7. for filtration and statistical analyses. Contaminants were removed from the dataset based on a contaminants database downloaded with the MaxQuant software, Max Planck Institute of Biochemistry, Martinsried, Germany. TMT abundances were log2 transformed. Technical replicates were averaged by mean. Proteins that were not quantified in all the 10 samples were excluded from the dataset. At least 2 unique peptides were required for successful identification. A paired *t*-test was conducted in Perseus using the FDR method by Benjamini and Hochberg [49]. The S_0_ constant in Perseus was set to 2. A protein was considered statistically significantly changed if *p* < 0.05, *FDR* < 0.01 and fold change >4.0 or fold change <0.25. Prior to calculation of fold changes, the log2 transformation of TMT values was reversed. Fold changes were then calculated as the average ratio of TMT abundance right eye/TMT abundance left eye. A PCA was conducted in Perseus with an *FDR* < 0.01 according to the method of Benjamini and Hochberg [49].

### 4.6. Network Analysis

Bioinformatic analyses were conducted using the STRING database (version 10.5) [50]. Upregulated proteins and downregulated proteins were analyzed as two individual groups. The multiple proteins function was selected in STRING. The background organism was set to *Homo sapiens* and UniProt *Homo sapiens* accession numbers of the differentially changed proteins were used. The minimum required interaction score was set to 0.700, corresponding to high confidence. For cluster analysis, the Markov Cluster algorithm was used with an inflation parameter set to 4. STRING was also used to perform Gene Ontology and KEGG pathways analyses with a Fisher’s exact test corrected by the *FDR* method of Benjamini and Hochberg [49,51].

### 4.7. Western Blotting

Western blotting was performed as previously described [13] using a primary monoclonal mouse anti-β-actin antibody 1:5000 (clone AC-15, Sigma-Aldrich, St. Louis, MO, USA), a primary polyclonal rabbit anti-IL-18 antibody 1:500 (MBS2026569, MyBioSource, San Diego, CA, USA), and a primary polyclonal rabbit anti-S100A12 antibody 1:100 (MBS2026249, MyBioSource, San Diego, CA, USA), diluted in 2.5% (*w*/*v*) skim milk blocking buffer. Log transformed densitometric data was used to perform a paired *t*-test.

### 4.8. Immunohistochemistry

Eyes from one animal were used for immunohistochemistry. Dissection and fixation were performed as previously described [13]. Hematoxylin and eosin staining was performed as described by Kiernan [52]. Anti-bodies used for staining included a polyclonal IgG antibody directed at IL-18 (MBS2026569, MyBioSource, San Diego, CA, USA), a polyclonal IgG antibody directed at S100A12 (MBS2026249, MyBioSource, San Diego, CA, USA), a polyclonal IgG antibody directed at ANXA1 (MBS2001804, MyBioSource, San Diego, CA, USA), a poly-clonal IgG antibody directed at fibronectin (ab23751, Abcam, Cambridge, UK), and a monoclonal IgG1 antibody directed at rhodopsin (ab190307, Abcam, Cambridge, UK). The antibodies were diluted in (1:200–1:800) in PBS + 0.3% Triton X100. The sections incubated overnight at 4 °C and were processed with EnVission (DakoCytomation) DAB. Controls were incubated with rabbit IgG_2b_ or irrelevant rabbit anti-bodies.

## 5. Conclusions

Proteome changes were studied in an RVO model that best corresponded to CRVO. This RVO model was complicated by retinal ischemia. RVO resulted in a downregulation of proteins involved in visual perception, including rhodopsin, transducin alpha subunit, transducin gamma chain, and long-wave sensitive opsin. The decreased contents of these photoreceptor proteins may represent an early stage of retinal degeneration. The decreased levels of GAD1 and GAD2 may indicate that neurotransmission is compromised in ischemic RVO, while downregulation of complexins 2–4 may affect synaptic fusion and neurotransmitter release. RVO was associated with increased retinal contents of IL-18, S100A12, and ANXA1. IL-18, and S100A12 may be important driving forces of inflammatory processes caused by ischemia. Additional studies will be required to further establish the roles of IL-18 and S100A12 in RVO. The potential of IL-18 and S100A12 as therapeutic targets may be addressed in future studies once the roles of these proteins in RVO have been established.

## Figures and Tables

**Table 1:** Proteins involved in visual perception that were downregulated in experimental retinal vein occlusion (RVO).

UniProt Accession	Gene Name	RVO/Control	*p*-Value	Protein Name
O18766	RHO	0.15	0.000278	Rhodopsin
Q7YS78	ARR3	0.15	1.80 × 10^−7^	Arrestin-C
P29973-2	CNGA1	0.15	0.000027	Isoform 2 of cGMP-gated cation channel alpha-1
P43080	GUCA1A	0.17	0.000056	Guanylyl cyclase-activating protein 1
P51160	PDE6C	0.19	7.73 × 10^−7^	Cone cGMP-specific 3′,5′-cyclic phosphodiesterase
P23942	PRPH2	0.2	0.0003223	Peripherin-2
P04000	OPN1LW	0.2	0.000014	Long-wave-sensitive opsin 1
P11488	GNAT1	0.23	0.000019	Transducin alpha-1 chain

**Table 2:** Proteins involved in neurotransmitter transport that were downregulated in RVO.

UniProt Accession	Gene Name	RVO/Control	*p*-Value	Protein Name
Q6PUV4	CPLX2	0.13	0.000077	Complexin-2
P46410	GLUL	0.13	0.0000056	Glutamine synthetase
P48321	GAD2	0.17	0.000014	Glutamate decarboxylase 2
Q9Y2J	RPH3A	0.18	0.000013	Rabphilin-3A
O14910	LIN7A	0.2	0.00014	Protein lin-7 homolog A
Q8WVH0	CPLX3	0.22	0.00020	Complexin-3
Q3I5G7	SNCA	0.22	0.00032	Alpha-synuclein
P48319	GAD1	0.22	0.000026	Glutamate decarboxylase 1
Q7Z7G2	CPLX4	0.23	0.000042	Complexin-4
O94925	GLS	0.23	0.000023	Glutaminase kidney isoform
Q96E17	RAB3C	0.24	0.000039	Ras-related protein Rab-3

**Table 3:** Proteins involved in inflammatory response that were upregulated in RVO.

UniProt Accession	Gene Name	RVO/Control	*p*-Value	Protein Name
P14287	SPP1	15.59	0.000069	Osteopontin
P29700	AHSG	11.95	0.00044	Alpha-2-HS-glycoprotein
P30034	PF4	11.57	0.0015	Platelet factor 4
P02671-1	FGA	11.49	0.00031	Fibrinogen alpha chain
P07996	THBS1	11.38	0.00087	Thrombospondin-1
P02751	FN1	10.9	0.000099	Fibronectin
P0C0L4-1	C4A	10.78	0.00064	Complement C4-A
Q2VL90	CD163	9.64	0.000056	Scavenger receptor cysteine-rich type 1 protein M130
P79263	ITIH4	8.9	0.0014	Inter-alpha-trypsin inhibitor heavy chain H4
P19619	ANXA1	8.16	0.000074	Annexin A1
P50447	SERPINA1	7.89	0.0011	Alpha-1-antitrypsin
Q8SPS7	HP	7.88	0.0023	Haptoglobin
Q19AZ8	F2	7.79	0.00069	Prothrombin
O19073	IL18	6.91	0.000022	Interleukin-18
P01025	C3	6.79	0.00076	Complement C3
P32394	HMOX1	6.69	0.000025	Heme oxygenase 1
P12068	LYZ	5.36	0.00055	Lysozyme c-2
P80310	S100A12	5.12	0.00018	S100-A12
P38571	LIPA	4.46	0.00024	Lysosomal acid lipase/cholesteryl ester hydrolase

## Supplementary Materials

Supplementary materials can be found at http://www.mdpi.com/1422-0067/19/11/3328/s1. Mass spectrometry raw data was uploaded to ProteomeXchange. Supplementary File S1—mass spectrometry data after filtering. Supplementary File 2—unfiltered mass spectrometry data. Supplementary File 3—list of all statistically significantly changed proteins. Supplementary File 4—cluster analysis of upregulated proteins. Supplementary File 5—biological processes of upregulated clustering proteins. Supplementary File 6—immunohistochemical staining for fibronectin.

## Abbreviations

ANXA1	Annexin A1
CNGA1	Isoform 2 of cGMP-gated cation channel alpha-1
CRVO	Central retinal vein occlusion
HE FDR	Hematoxylin and eosin False-discovery rate
GAD1	Glutamate decarboxylase 1
GAD2	Glutamate decarboxylase 2
GABA	Gamma-aminobutyric acid
GCAP1	Guanylyl cyclase-activating protein 1
GCL	Ganglion cell layer
IL-18	Interleukin-18
ILM IPL	Inner limiting membrane Inner plexiform layer
IS	Photoreceptor inner segment
ONL	Outer nuclear layer
OPL	Outer plexiform layer
OS	Photoreceptor outer segment
PCA	Principal component analysis
PDE6C	Cone cGMP-specific 3′,5′-cyclic phosphodiesterase
RPE RVO	Retinal pigment epithelium Retinal vein occlusion
TEAB	Triethyl ammonium bicarbonate
TMT	Tandem mass tags
VEGF	Vascular endothelial growth factor
