# Supplementary figures and images for: IL-18 and S100A12 Are Upregulated in Experimental Central Retinal Vein Occlusion
Source: Int J Mol Sci. 2018 Oct 25;19(11):3328. doi: 10.3390/ijms19113328 (PMC6274751; doi:10.3390/ijms19113328)

# Upregulated proteins - blood coagulation

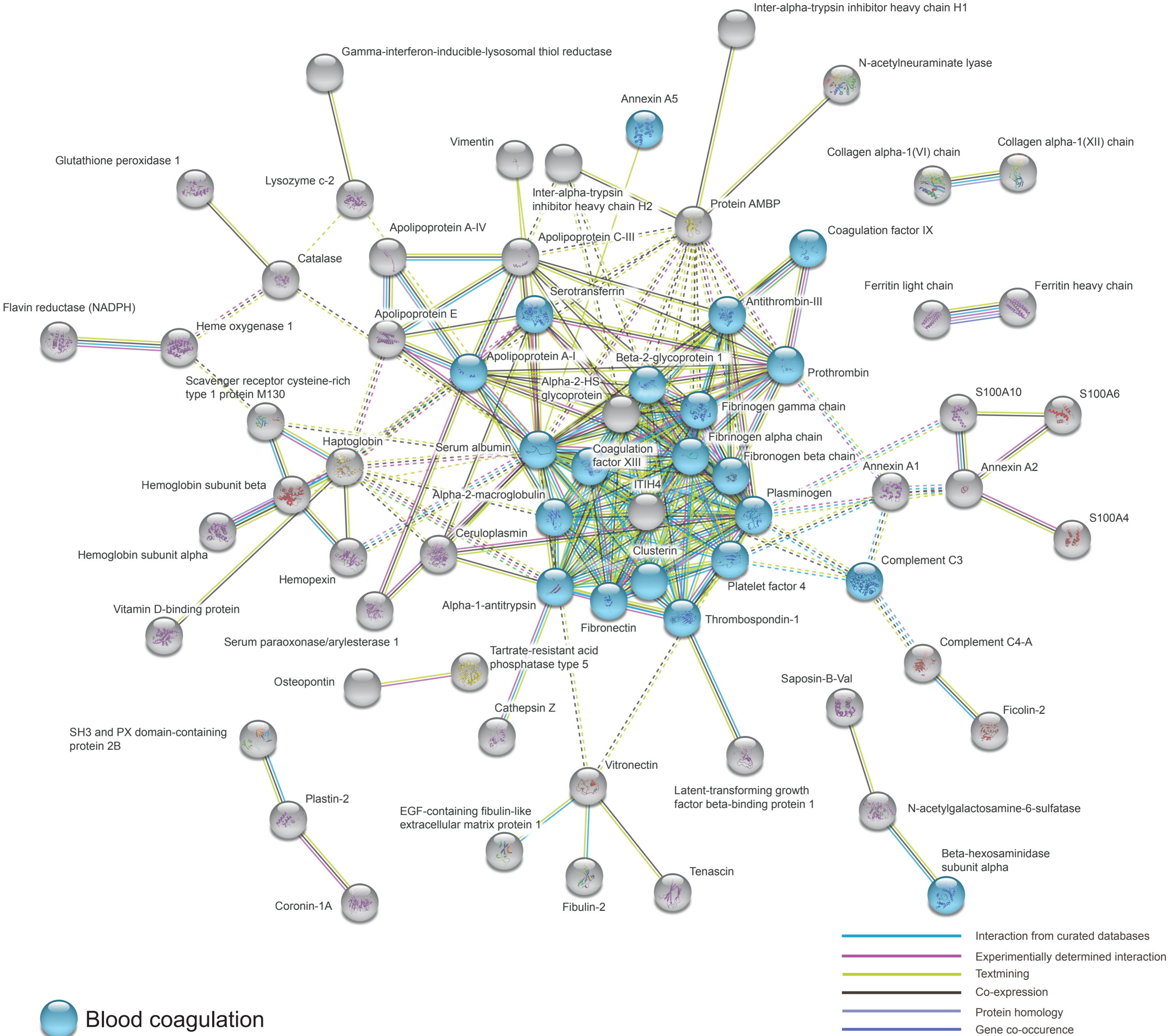

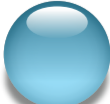 Blood coagulation

Supplement: Supplementary file 1 [file ijms-19-03328-s001.zip › Supplementary file 5.pdf]

Fibronectin  
RVO

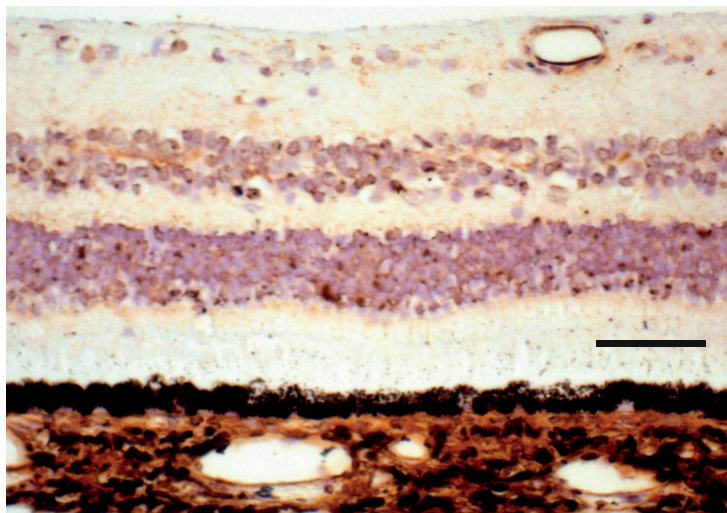

Fibronectin  
Control laser

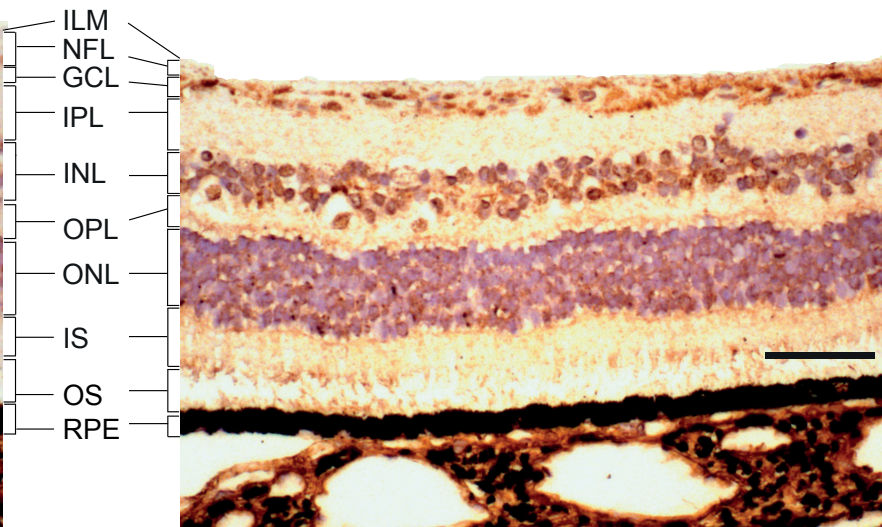

ILM  
NFL  
GCL  
IPL  
INL  
OPL  
ONL  
IS  
OS  
RPE

Supplement: Supplementary file 1 [file ijms-19-03328-s001.zip › Supplementary file 6.pdf]

# Cluster analysis of upregulated proteins

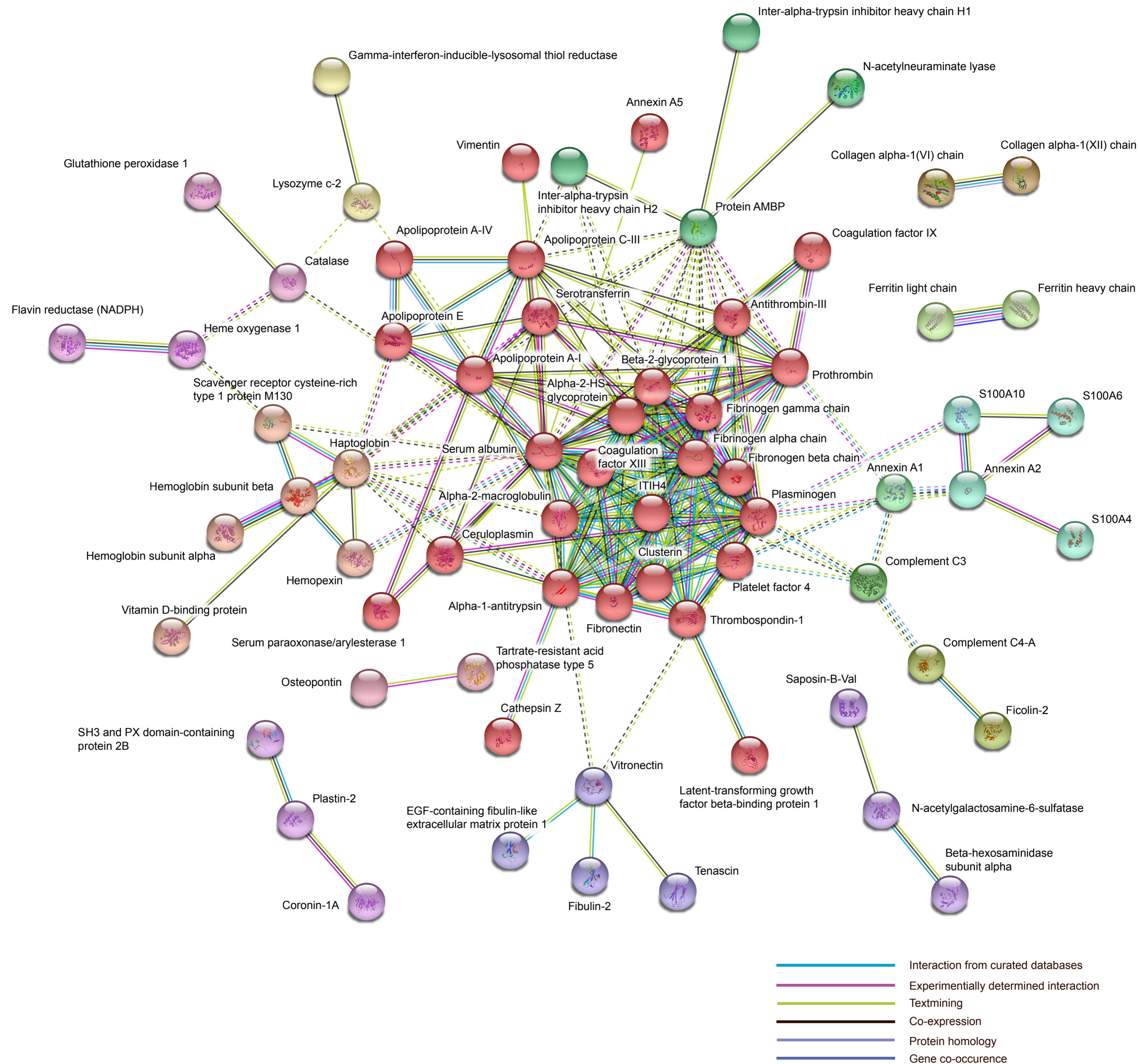

Supplement: Supplementary file 1 [file ijms-19-03328-s001.zip › Supplementary file 4.pdf]
