# Supplementary material for: IL-18 and S100A12 Are Upregulated in Experimental Central Retinal Vein Occlusion
Source: Int J Mol Sci. 2018 Oct 25;19(11):3328. doi: 10.3390/ijms19113328 (PMC6274751; doi:10.3390/ijms19113328)
Supplement: Supplementary file 1 [file ijms-19-03328-s001.zip › Supplementary file 3.doc]

| **UniProt**  **accession no.** | **Gene name** | **Ratio CRVO /**  **control** | **p-value** | **Protein name** |
| --- | --- | --- | --- | --- |
| P02679 | FGG | 51.70 | 0.00085 | Fibrinogen gamma chain |
| P19130 | FTH1 | 21.89 | 0.00030 | Ferritin heavy chain |
| Q9BXX0 | EMILIN2 | 19.21 | 0.0013 | EMILIN-2 |
| P00488 | F13A1 | 17.31 | 0.00023 | Coagulation factor XIII A chain |
| P24853 | IGFBP2 | 17.24 | 0.00092 | Insulin-like growth factor-binding protein 2 |
| P02749 | APOH | 15.88 | 0.00056 | Beta-2-glycoprotein 1 |
| P14287 | SPP1 | 15.59 | 0.000069 | Osteopontin |
| P02774-3 | GC | 14.59 | 0.00090 | Isoform 3 of Vitamin D-binding protein |
| P02067 | HBB | 12.89 | 0.0015 | Hemoglobin subunit beta |
| P01965 | HBA | 12.85 | 0.0013 | Hemoglobin subunit alpha |
| P04366 | AMBP | 12.67 | 0.000050 | Protein AMBP |
| P16293 | F9 | 12.2 | 0.00035 | Coagulation factor IX |
| P29700 | AHSG | 11.95 | 0.00044 | Alpha-2-HS-glycoprotein |
| P02675 | FGB | 11.79 | 0.00039 | Fibrinogen beta chain |
| P48819 | VTN | 11.79 | 0.00017 | Vitronectin |
| P30034 | PF4 | 11.57 | 0.0015 | Platelet factor 4 |
| P00450 | CP | 11.51 | 0.00037 | Ceruloplasmin |
| P02671-1 | FGA | 11.49 | 0.00031 | Fibrinogen alpha chain |
| P07996 | THBS1 | 11.38 | 0.00087 | Thrombospondin-1 |
| P02751 | FN1 | 10.9 | 0.000099 | Fibronectin |
| Q92743 | HTRA1 | 10.83 | 0.00012 | Serine protease HTRA1 |
| P0C0L4-1 | C4A | 10.78 | 0.00064 | Complement C4-A |
| P02549-1 | SPTA1 | 10.65 | 0.0012 | Spectrin alpha chain. erythrocytic 1 |
| P08835 | ALB | 10.57 | 0.00031 | Serum albumin |
| Q29041 | FCN2 | 10.47 | 0.00028 | Ficolin-2 |
| Q29549 | CLU | 10.29 | 0.0000060 | Clusterin |
| Q14766-4 | LTBP1 | 10.04 | 0.00013 | Isoform 4 of Latent-transforming growth factor beta-binding protein 1 |
| Q15582 | TGFBI | 9.95 | 0.00025 | Transforming growth factor-beta-induced protein ig-h3 |
| O00160 | MYO1F | 9.7 | 0.0000084 | Unconventional myosin-If |
| Q2VL90 | CD163 | 9.64 | 0.000056 | Scavenger receptor cysteine-rich type 1 protein M130 |
| P01846 |  | 9.61 | 0.00046 | Ig lambda chain C region |
| Q99715 | COL12A1 | 9.54 | 0.00010 | Collagen alpha-1(XII) chain |
| O02668 | ITIH2 | 9.51 | 0.00014 | Inter-alpha-trypsin inhibitor heavy chain H2 |
| P27169 | PON1 | 9.5 | 0.00076 | Serum paraoxonase/arylesterase 1 |
| P06867 | PLG | 9.47 | 0.00020 | Plasminogen |
| B3SP85 | IFI30 | 9.01 | 0.000067 | Gamma-interferon-inducible-lysosomal thiol reductase |
| P01023 | A2M | 8.96 | 0.00025 | Alpha-2-macroglobulin [OS=Homo sapiens] |
| Q12805 | EFEMP1 | 8.95 | 0.000056 | EGF-containing fibulin-like extracellular matrix protein 1 |
| P79263 | ITIH4 | 8.9 | 0.0014 | Inter-alpha-trypsin inhibitor heavy chain H4 |
| P19133 | FTL | 8.85 | 0.00017 | Ferritin light chain |
| P18648 | APOA1 | 8.35 | 0.00030 | Apolipoprotein A-I |
| P19619 | ANXA1 | 8.16 | 0.000074 | Annexin A1 |
| Q9GK37 | SERPINA6 | 8.15 | 0.00014 | Corticosteroid-binding globulin |
| O94788-1 | ALDH1A2 | 8.02 | 0.00079 | Retinal dehydrogenase 2 |
| P18650 | APOE | 7.97 | 0.00025 | Apolipoprotein E |
| P50447 | SERPINA1 | 7.89 | 0.0011 | Alpha-1-antitrypsin |
| Q8SPS7 | HP | 7.88 | 0.0023 | Haptoglobin |
| O46409 | APOA4 | 7.85 | 0.00038 | Apolipoprotein A-IV |
| P27917 | APOC3 | 7.81 | 0.0012 | Apolipoprotein C-III |
| Q19AZ8 | F2 | 7.79 | 0.00069 | Prothrombin |
| Q2EN75 | S100A6 | 7.34 | 0.000022 | Protein S100-A6 |
| P81405 | PSAP | 7.3 | 0.0029 | Saposin-B-Val |
| O46427 | CTSH | 7.3 | 0.000017 | Pro-cathepsin H |
| P04040 | CAT | 7.12 | 0.0019 | Catalase |
| Q6YHK3-1 | CD109 | 7.11 | 0.000082 | CD109 antigen |
| P98095-2 | FBLN2 | 6.92 | 0.00059 | Isoform 2 of Fibulin-2 |
| O19073 | IL18 | 6.91 | 0.000022 | Interleukin-18 |
| Q9UBR2 | CTSZ | 6.83 | 0.000020 | Cathepsin Z |
| P01025 | C3 | 6.79 | 0.00076 | Complement C3 |
| P32394 | HMOX1 | 6.69 | 0.000025 | Heme oxygenase 1 |
| P09889 | ACP5 | 6.68 | 0.00013 | Tartrate-resistant acid phosphatase type 5 |
| Q14315 | FLNC | 6.63 | 0.000013 | Filamin-C |
| O62839 | CAT | 6.62 | 0.0013 | Catalase |
| Q03472 | C4BPA | 6.61 | 0.00018 | Apolipoprotein R |
| P13796 | LCP1 | 6.49 | 0.000027 | Plastin-2 |
| P08758 | ANXA5 | 6.44 | 0.00037 | Annexin A5 |
| O97763 | NPC2 | 6.37 | 0.00013 | Epididymal secretory protein E1 |
| P79281 | PTN | 6.33 | 0.00051 | Pleiotrophin |
| P50479-1 | PDLIM4 | 6.3 | 0.0000024 | PDZ and LIM domain protein 4 |
| P30043 | BLVRB | 6.3 | 0.0012 | Flavin reductase (NADPH) |
| P50828 | HPX | 6.23 | 0.0017 | Hemopexin |
| O15143 | ARPC1B | 6.1 | 7.59 x 10^-8^ | Actin-related protein 2/3 complex subunit 1B |
| P08582-1 | MELTF | 5.92 | 0.000299 | Melanotransferrin |
| P35443 | THBS4 | 5.87 | 0.0034 | Thrombospondin-4 |
| Q7M2W6 | CRYAB | 5.83 | 0.00015 | Alpha-crystallin B chain |
| P09571 | TF | 5.77 | 0.00087 | Serotransferrin |
| P48681 | NES | 5.62 | 0.00044 | Nestin |
| P12109 | COL6A1 | 5.58 | 0.00016 | Collagen alpha-1(VI) chain |
| P01008 | SERPINC1 | 5.51 | 0.0023 | Antithrombin-III |
| Q9BU40-4 | CHRDL1 | 5.43 | 0.000078 | Isoform 4 of Chordin-like protein 1 |
| P12068 | LYZ | 5.36 | 0.00055 | Lysozyme c-2 |
| Q29116-2 | TNC | 5.34 | 0.00020 | Isoform Major of Tenascin |
| Q29052 | ITIH1 | 5.28 | 0.00066 | Inter-alpha-trypsin inhibitor heavy chain H1 |
| Q14764 | MVP | 5.16 | 0.000022 | Major vault protein |
| P26447 | S100A4 | 5.13 | 0.000025 | Protein S100-A4 |
| P80310 | S100A12 | 5.12 | 0.00018 | Protein S100-A12 |
| P19620 | ANXA2 | 5.1 | 0.00013 | Annexin A2 |
| P17931 | LGALS3 | 4.96 | 0.0000037 | Galectin-3 |
| P15468 | RNASE4 | 4.92 | 0.000065 | Ribonuclease 4 |
| P29350-4 | PTPN6 | 4.87 | 0.000014 | Isoform 4 of Tyrosine-protein phosphatase non-receptor type 6 |
| Q96HF1 | SFRP2 | 4.83 | 0.000067 | Secreted frizzled-related protein 2 |
| Q9BEC7 | NPL | 4.81 | 0.0028 | N-acetylneuraminate lyase |
| A7VK00 | MX2 | 4.77 | 0.0042 | interferon-induced GTP-binding protein Mx2 |
| A2TLM1 | RPIA | 4.55 | 0.00038 | ribose-5-phosphate isomerase |
| A1X283 | SH3PXD2B | 4.55 | 0.000049 | SH3 and PX domain-containing protein 2B |
| Q8WNQ7 | GALNS | 4.48 | 4.6 x 10^-7^ | N-acetylgalactosamine-6-sulfatase |
| P60903 | S100A10 | 4.48 | 0.000042 | Protein S100-A10 |
| P38571 | LIPA | 4.46 | 0.00024 | Lysosomal acid lipase/cholesteryl ester hydrolase |
| P06865 | HEXA | 4.44 | 0.0000034 | Beta-hexosaminidase subunit alpha |
| O95084 | PRSS23 | 4.42 | 0.00088 | Serine protease 23 |
| P31146 | CORO1A | 4.4 | 0.0000014 | Coronin-1A |
| Q29545 | ICA | 4.35 | 0.0015 | Inhibitor of carbonic anhydrase |
| Q8MJ14 | GPX1 | 4.32 | 0.000050 | Glutathione peroxidase 1 |
| P14632 | LTF | 4.23 | 0.0012 | Lactotransferrin |
| P14317 | HCLS1 | 4.13 | 0.000072 | Hematopoietic lineage cell-specific protein |
| P02543 | VIM | 4.01 | 0.000062 | Vimentin |
| Q8IUA7-1 | ABCA9 | 0.24 | 0.000038 | ATP-binding cassette sub-family A member 9 |
| Q96E17 | RAB3C | 0.24 | 0.000039 | Ras-related protein Rab-3C |
| Q9TSV6 | ATP6V1G2 | 0.24 | 0.000034 | V-type proton ATPase subunit G 2 |
| O94811 | TPPP | 0.24 | 0.00019 | Tubulin polymerization-promoting protein |
| P11488 | GNAT1 | 0.23 | 0.000019 | Guanine nucleotide-binding protein G(t) subunit alpha-1 |
| O94925-1 | GLS | 0.23 | 0.000023 | Glutaminase kidney isoform. mitochondrial |
| Q7Z7G2 | CPLX4 | 0.23 | 0.000042 | Complexin-4 |
| P48319 | GAD1 | 0.22 | 0.000026 | Glutamate decarboxylase 1 |
| Q8WVH0 | CPLX3 | 0.22 | 0.00020 | Complexin-3 |
| Q3I5G7 | SNCA | 0.22 | 0.00032 | Alpha-synuclein |
| B1AK53-1 | ESPN | 0.22 | 0.000026 | Espin |
| Q8IVF2-1 | AHNAK2 | 0.21 | 0.000046 | Protein AHNAK2 |
| P00506 | GOT2 | 0.2 | 0.0000046 | Aspartate aminotransferase. mitochondrial |
| O14910 | LIN7A | 0.2 | 0.00014 | Protein lin-7 homolog A |
| P04000 | OPN1LW | 0.2 | 0.000014 | Long-wave-sensitive opsin 1 |
| P12036 | NEFH | 0.2 | 0.000050 | Neurofilament heavy polypeptide |
| P23942 | PRPH2 | 0.2 | 0.00032 | Peripherin-2 [OS=Homo sapiens] |
| P63211 | GNGT1 | 0.2 | 0.0000070 | Guanine nucleotide-binding protein G(T) subunit gamma-T1 |
| Q16143 | SNCB | 0.19 | 0.000024 | Beta-synuclein |
| Q05193-2 | DNM1 | 0.19 | 0.00029 | Isoform 2 of Dynamin-1 |
| Q96JB8-1 | MPP4 | 0.19 | 0.000034 | MAGUK p55 subfamily member 4 |
| P51160 | PDE6C | 0.19 | 7.73E-07 | Cone cGMP-specific 3'.5'-cyclic phosphodiesterase subunit alpha' |
| Q14576 | ELAVL3 | 0.18 | 0.000055 | ELAV-like protein 3 |
| Q9UK22 | FBXO2 | 0.18 | 0.000069 | F-box only protein 2 |
| P30531 | SLC6A1 | 0.18 | 0.000056 | Sodium- and chloride-dependent GABA transporter 1 |
| Q9Y2J0-1 | RPH3A | 0.18 | 0.000013 | Rabphilin-3A |
| P48321 | GAD2 | 0.17 | 0.000014 | Glutamate decarboxylase 2 |
| P43080 | GUCA1A | 0.17 | 0.000056 | Guanylyl cyclase-activating protein 1 |
| P07196 | NEFL | 0.17 | 0.000030 | Neurofilament light polypeptide |
| P07197 | NEFM | 0.16 | 0.000017 | Neurofilament medium polypeptide |
| P02547 | NEFL | 0.16 | 0.000027 | Neurofilament light polypeptide |
| P57771-2 | RGS8 | 0.16 | 0.000063 | Isoform 2 of Regulator of G-protein signaling 8 |
| P10745 | RBP3 | 0.15 | 0.0000074 | Retinol-binding protein 3 |
| P29973-2 | CNGA1 | 0.15 | 0.000027 | Isoform 2 of cGMP-gated cation channel alpha-1 |
| Q7YS78 | ARR3 | 0.15 | 1.80 x 10^-7^ | Arrestin-C |
| O14810 | CPLX1 | 0.15 | 0.00011 | Complexin-1 |
| O18766 | RHO | 0.15 | 0.00028 | Rhodopsin |
| Q9P2W3 | GNG13 | 0.13 | 0.00015 | Guanine nucleotide-binding protein G(I)/G(S)/G(O) subunit gamma-13 |
| Q6PUV4 | CPLX2 | 0.13 | 0.000077 | Complexin-2 |
| P46410 | GLUL | 0.13 | 0.000056 | Glutamine synthetase |
| P08552 | NEFM | 0.12 | 0.000085 | Neurofilament medium polypeptide |
